# Convergently evolved placental villi show multiscale structural adaptations to differential placental invasiveness

**DOI:** 10.1098/rsbl.2024.0016

**Published:** 2024-03-27

**Authors:** Davis Laundon, Bram G. Sengers, James Thompson, Shelley E. Harris, Olivia Beasley, Philip J. Basford, Orestis L. Katsamenis, Patricia Goggin, Emilie Derisoud, Diana Fanelli, Carlotta Bocci, Francesco Camillo, Justine Shotton, Georgina Constable-Dakeyne, Neil J. Gostling, Pascale Chavatte-Palmer, Rohan M. Lewis

**Affiliations:** ^1^ The Institute of Developmental Sciences, Human Development and Health, Faculty of Medicine, University of Southampton, Southampton SO16 6YD, UK; ^2^ Institute for Life Sciences, University of Southampton, University Road, Highfield, Southampton SO17 1BJ, UK; ^3^ School of Engineering, Faculty of Engineering and Physical Sciences, University of Southampton, University Road, Southampton SO17 1BJ, UK; ^4^ Biomedical Imaging Unit, Faculty of Medicine, University of Southampton, Southampton SO16 6YD, UK; ^5^ School of Biological Sciences, Faculty of Environmental and Life Sciences, University of Southampton, University Rd, Highfield, Southampton SO17 1BJ, UK; ^6^ μ-VIS X-Ray Imaging Centre, Faculty of Engineering and Physical Sciences, University of Southampton, Southampton SO17 1BJ, UK; ^7^ Université Paris-Saclay, UVSQ, INRAE, BREED, 78350 Jouy-en-Josas, France; ^8^ Ecole Nationale Vétérinaire d'Alfort, BREED, 94700 Maisons-Alfort, France; ^9^ Dipartimento di Scienze Veterinarie, Università di Pisa, Via Livornese lato monte, 56121 San Piero a Grado, Pisa, Italy; ^10^ Marwell Wildlife, Thompson's Lane, Colden Common, Winchester SO21 1JH, UK

**Keywords:** placenta, evolution, microCT, microscopy, structure

## Abstract

Despite having a single evolutionary origin and conserved function, the mammalian placenta exhibits radical structural diversity. The evolutionary drivers and functional consequences of placental structural diversity are poorly understood. Humans and equids both display treelike placental villi, however these villi evolved independently and exhibit starkly different levels of invasiveness into maternal tissue (i.e. the number of maternal tissue layers between placental tissue and maternal blood). The villi in these species therefore serve as a compelling evolutionary case study to explore whether placentas have developed structural adaptations to respond to the challenge of reduced nutrient availability in less invasive placentas. Here, we use three-dimensional X-ray microfocus computed tomography and electron microscopy to quantitatively evaluate key structures involved in exchange in human and equid placental villi. We find that equid villi have a higher surface area to volume ratio and deeper trophoblastic vessel indentation than human villi. Using illustrative computational models, we propose that these structural adaptations have evolved in equids to boost nutrient transfer to compensate for reduced invasiveness into maternal tissue. We discuss these findings in relation to the ‘maternal–fetal conflict hypothesis’ of placental evolution.

## 1. Introduction

Placental function underpins fetal development and postnatal health by facilitating physiological exchange between the mother and fetus [1]. The placenta enables the transfer of nutrients and oxygen to the fetus from the mother and removes waste products from the fetal bloodstream, as well as acting as a barrier to toxins and pathogens [2]. The mammalian placenta is an ephemeral fetal organ which, despite having a single evolutionary origin and conserved functions, exhibits massive morphological and structural diversity across the group [3–5]. The evolutionary pressures governing morphological divergence, and how structure interacts with physiological outcomes, are poorly understood.

Mammalian placentas are grouped into so-called ‘placental types’ [3,4,6–8] according to the way in which maternal and fetal tissues interact (placental interdigitation) and the number of tissue layers separating maternal and fetal circulations (placental invasiveness). Different placental types have been found to correlate with life-history strategies [9], environmental parasite diversity [10] and conflict between maternal and fetus fitness interests [11], however the picture is far from clear. Grouping placental structural variation into qualitative categories may obscure the importance of underlying structural differences across different scales that impact placental function and reproductive success [8].

**Figure 1.**
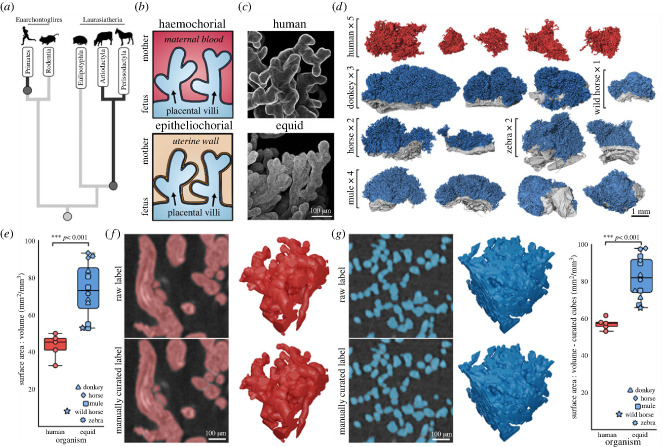
Equid placental villi have a higher surface area to volume ratio (SA : V) than human villi. (*a*) Placental villi evolved independently in humans and equids (dark lines and circles) from an ancestral labyrinthine placenta (light circle). Simplified tree from the phylogeny outlined in [12]. (*b*) Human placentas are haemochorial, where villi are in direct contact with maternal blood, whereas equid placentas are epitheliochorial, where villi are only in contact with the maternal uterine epithelium. (*c*) Fluorescent confocal microscope images of lectin-stained (DSL) human and equid (horse) villi highlighting qualitative differences in morphology. Images are maximum intensity z-projections. (*d*) Three-dimensional reconstructions from microCT imaging of human (red) and equid (blue) placental villi samples used in this study. Also shown in the equid reconstructions is the non-villous portion of placental samples (grey). (*e*) MicroCT reconstructions revealed that equid placental villi have a higher SA : V ratio than human placental villi. (*f,g*) Subvolumes for (*f*) human and (*g*) equid (horse) microCT villi were scaled and manually curated to separate connected components. Shown are two-dimensional slices from the volumes (left) and the corresponding three-dimensional reconstruction (right). (*h*) Equid placental villi were again found to have a higher SA : V than human villi in the curated subvolumes. Animal silhouettes from PhyloPic https://www.phylopic.org/.

Human and equid (horses and their kin) placentas are both ‘villous’ placental types, whereby interdigitation with maternal tissue is through treelike branching placental ‘villi’ (figure 1*a–c*) [7], however their villi evolved independently (figure 1*a*). Despite showing superficially similar interdigitation, human and equid placentas display very different levels of invasiveness into maternal tissue (figure 1*b*). In humans, the placenta is ‘haemochorial’ whereby the outer cellular layer of the placental villi (the villous trophoblast) is directly bathed in maternal blood (high invasiveness). By contrast, in equids the placenta is ‘epitheliochorial’ whereby the villous trophoblast is only in contact with the maternal uterine epithelium (low invasiveness). Epitheliochorial placentas are thought to permit less efficient transfer of nutrients from mother to fetus than haemochorial placentas, due to the additional tissue layers between maternal and fetal circulation acting as barriers to transfer [8]. Ancestral trait reconstruction suggests that the ancestral placenta in placental mammals was haemochorial, therefore the epitheliochorial placenta is the derived condition [3,13–16]. Evolutionary pressures proposed to favour the origin of a less invasive placenta include protection of the mother from oxidative stress by fetal metabolism [17], protection of the fetus from vertical transmission of parasites from the mother [10], and the increased ability for the mother to withhold nutrients from the fetus in a phenomenon known as ‘maternal constraint’ [18,19].

Maternal constraint is particularly important when understood in relation to the ‘maternal–fetal conflict hypothesis' in placental evolution. According to this hypothesis, the long-term fitness interests of the mother and fetus can become misaligned and the resulting conflict has been proposed to present a key driver in placental evolution [11,20–22]. For example, according to the conflict hypothesis, fetuses with highly invasive placentas could extract more nutrients from the mother than it is optimal for her to give, benefitting fetal fitness in terms of increased growth of the current fetus at the expense of the mother's future reproduction. In species with large precocial offspring which demand high levels of energy investment, such as horses, additional maternal tissue layers may have evolved to allow the mother to constrain the transfer of nutrients to any given offspring to benefit her overall lifetime fitness [19,23]. The pressure for constraint would not be as strong in species with more altricial young, where energy investment is relatively lower. The extent to which the placenta would structurally respond to a reduction in invasiveness and nutrient availability is not known.

Do placentas show structural adaptations to compensate for reduced invasiveness into maternal tissue? This question gains importance when considering that human and equid placentas, despite displaying superficially similar villous interdigitation, differ significantly in placental invasiveness. Their comparison represents a powerful evolutionary case study to explore this idea. Here, we used 3D X-ray microfocus computed tomography (microCT) and electron microscopy (EM) to structurally quantify placental villi from humans and equids across scales. We hypothesized that, given the epitheliochorial organization of the equid placenta, equid villi would display structural adaptations to boost nutrient transfer in response to reduced placental invasiveness relative to humans at different scales.

## 2. Methods

### (a) Sample collection and ethics

Human (*Homo sapiens*) placentas (*n* = 5) were collected following delivery from uncomplicated pregnancies with written informed consent and ethical approval from the Southampton and Southwest Hampshire Local Ethics Committee (11/SC/0529). Equid placentas (*n* = 12) were collected non-invasively after foaling at spontaneous placental delivery from healthy full-term pregnancies as part of routine placental checks. Saddlebred horse (*Equus caballus*) placentas (*n* = 2) were collected at the experimental farm of Institut Français du Cheval et de l'Equitation (IFCE), France. Grévy's zebra (*E. grevyi*) placentas (*n* = 2) and a Przewalski's wild horse (*E. ferus*) placenta (*n* = 1) were collected at Marwell Wildlife, UK. Donkey (*E. asinus*) placentas (*n =* 3) and mule (*E*. *asinus* × *E*. *caballus*) placentas (*n =* 4) were collected at the Department of Veterinary Sciences at Pisa University, Italy. Sample analysis in Southampton received local approval from the University of Southampton (47770.A1/46381.A2).

### (b) Sample processing and imaging

Small pieces of villous placental tissue (approximately 0.5 × 0.5 cm) were immediately fixed in 3% glutaraldehyde 0.1 M cacodylate buffer (pH 7.4) upon collection and stored at 4°C until sample processing. In equids, all samples were collected from the area <10 cm from the umbilical cord insertion, in the ‘body’ of the placenta, in order to standardize sampling. Samples were processed, imaged, and segmented according to the protocol detailed in [24]. Briefly, pieces of fixed placental tissue (approx. 3–5 mm) were dissected, heavy-metal stained (with osmium tetroxide, uranyl acetate and lead nitrate), dehydrated and embedded in Spurr's resin. The resin blocks were then scanned using a custom Nikon XTH 225 ST (Nikon X-Tek Systems Ltd) microCT scanner at the μ-VIS X-ray Imaging Centre, three-dimensional X-ray Histology Facility, University of Southampton (www.muvis.org) [25], generating image volumes of 4.8 µm isotropic voxels. Scanned blocks were then trimmed by ultramicrotomy using an EM UC7 ultramicrotome (Leica, Germany). Villous tissue subvolumes of interest identified from microCT datasets were excised from the resin blocks and mounted onto aluminium pins (TABB—G312/1) with conductive glue (CircuitWorks Conductive Epoxy CW2400; ITW Chemtronics). Pins were imaged by block face scanning EM using a 3View system (Gatan, Abingdon, UK) mounted in a Quanta250 Field Emission Gun Scanning Electron Microscope (FEG-SEM) (ThermoFisher, Eindhoven, The Netherlands). Two-dimensional cross sections of villi were randomly imaged (*n* ≥ 5 different villi per biological placenta replicate) across the block face at 15 nm XY pixels.

### (c) Image analysis and statistical comparison

Image datasets were imported into Microscopy Image Browser (MIB) [26] as .tiff stacks and segmented by intensity thresholding combined with semi-manual curation. This involved masking regions of interest and thresholding tissue within the bounds of the mask, as detailed in [24]. Labels were further statistically thresholded to clean up erroneous objects by excluding connected components less than 1 k voxels in three dimensions. Labels were quantified for volume, surface area and other morphometric parameters within MIB. Intensity thresholding of villi from microCT datasets resulted in segmented labels where individual villi were sometimes incorrectly connected in two-dimensional slices, however manual separation of every villus across whole datasets was prohibitive and watershed separation ineffective for these datasets. To eliminate the possibility that this connectivity affected accurate surface area quantification, 128 × 128 × 128 voxel subvolumes of villi were cropped from each sample and manually curated (figure 1*f,g*). The scaling differences between non-curated and curated subvolumes for villus volume (mean ± s.d., 0.96 ± 0.03) and surface area (1.11 ± 0.07) were extrapolated across the whole tissue volumes to correct for villus connectivity. These scaling differences were not significantly different for humans and equids (*p =* 0.35). Curated subvolumes were also independently compared between species (figure 1*h*).

Data were evaluated for prior normality and homogeneity assumptions with a Shapiro and Levene's test respectively. Morphometric differences between human and equid placental villi were evaluated using a *t*-test, or a Mann–Whitney *U*-test if the above assumptions were not met. The scipy package [27] run with Python 3.9.12 implemented in Jupyter Notebook 6.4.8 was used for all statistical comparisons. Means and standard deviations were calculated from mean values per biological placenta.

### (d) Computational modelling of nutrient transfer

Segmented images were imported into Simpleware ScanIP (P-2019.09; Synopsys, Inc., Mountain View, USA) for mesh generation. Steady state diffusion simulations were performed in COMSOL Multiphysics (v6.0; COMSOL AB, Stockholm, Sweden). Only normalized concentrations were considered, therefore boundary conditions of 1 and 0 mol m^−3^ were applied to the outer villus and inner capillary surface respectively. For facilitated diffusion, the normal flux *J*_BM_ (mol m^−2^ s^−1^) at the basal membrane was assumed to be governed by the following equation for a facilitative transporter [28]:

2.1 $$J_{\mathrm{BM}}=V_{\max}\left(\frac{c_{1}}{K_{m}+c_{1}}-\frac{c_{2}}{K_{m}+c_{2}}\right),$$

where *V*_max_ (mol m^−2^ s^−1^) is the maximum transport rate and *K*_m_ (mol m^−3^) the half maximum rate concentration, set to 0.5, while *c*_1_ is the local intracellular concentration in the trophoblast and *c*_2_ the local extracellular concentration in the stroma on either side of the basal membrane.

## 3. Results

**Figure 2.**
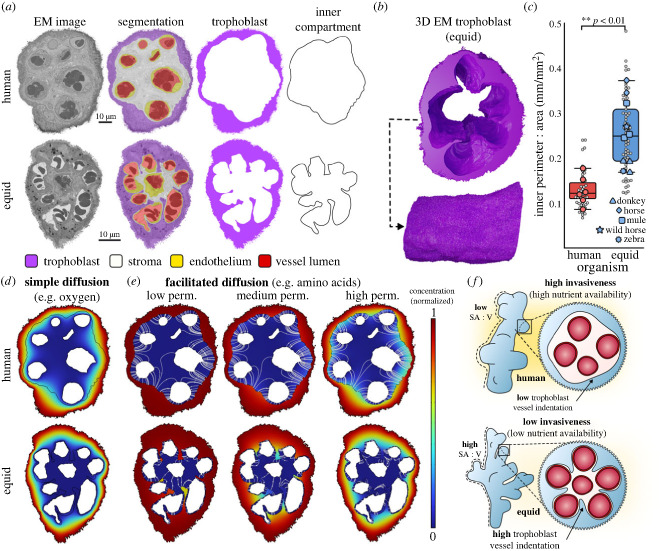
Equid placental villi display deep trophoblastic invaginations and a higher inner perimeter to area ratio (P : A) than human villi. (*a*) Illustrative example of a two-dimensional cross section through human and equid placental villi showing deep trophoblastic basal invaginations in equids but not in humans. (*b*) Three-dimensional serial block face scanning electron microscopy (SBF-SEM) reconstruction of the trophoblast showing basal invaginations into the stroma in an equid (horse) villus. (*c*) Electron microscopy of villus cross sections revealed that the inner compartment of equid placental villi has a higher P : A ratio than in human placental villi. Grey points indicate individual villus replicates (*n* ≥ 5 different villi per placenta), coloured points indicate means from each whole placenta. (*d,e*) Illustrative physiological modelling of simple diffusion (*d*) and facilitated (transporter-mediated) diffusion (*e*) through human and equid villus cross sections. Concentration gradients shown ranging from red (high) to blue (low). Facilitated diffusion was modelled at low, medium, and high permeabilities of the basal trophoblast membrane (*e*) with overlayed white lines showing illustrative routes of transfer. (*f*) Diagrammatic summary of the findings of this study.

MicroCT imaging allowed us to reconstruct and morphometrically quantify the villi of human and equid (donkey, wild horse, horse, zebra and mule) placentas (figure 1*d*). Quantification of the microCT reconstructions revealed that equid placental villi have a higher surface area to volume ratio (SA : V) (73.47 ± 14.91) than human villi (43.34 ± 6.78, *p* < 0.001) (figure 1*e*). This was also true when imaged villi were scaled to the same subvolumes and manually curated (figure 1*f,g*) (82.44 ± 11.54 versus 57.20 ± 3.13) (*p* < 0.001) (figure 1*h*). Plotting of individual equid placental replicates shows a non-overlapping distribution when compared with human samples, providing justification for the lumped ‘equid’ category in this study. (Figure 1*e,h*). At the scale of EM, equid placental villi displayed deep trophoblastic invaginations between blood vessels (figure 2*a,b*) resulting in deeper vascular indentation than in human villi. This can be quantified by calculating the perimeter to area ratio (P : A) of the inner villus compartment (blood vessels bound by stroma), which was higher in equids (0.24 ± 0.07) than in humans (0.12 ± 0.03) (*p* < 0.01) (figure 2*c*). This quantitative metric means that the inner compartment is in relatively higher contact with the basal membrane of the trophoblast in equids than in humans. Illustrative modelling of diffusive processes suggests that these protrusions are unlikely to influence simple transfer of gases such as oxygen (figure 2*d*) but may aid facilitated transporter-mediated transfer of substances such as amino acids (figure 2*e*). As transporter proteins are embedded in membrane boundaries between tissue layers, this is particularly true for modelled scenarios with low membrane permeabilities (figure 2*e*). It can also be observed that the deeper vessel indentation in equid than human villi produces shorter diffusion pathways from the trophoblast basal membrane to placental blood vessels, particularly for those in the villus centre (figure 2*e*).

## 4. Discussion

Using microCT and EM, we imaged and structurally quantified convergently evolved villi from human and equid placentas. We demonstrate that equid villi have a higher SA : V than human villi, and display basal trophoblastic invaginations which confer deeper blood vessel indentation. We propose that these multiscale structural differences may provide certain advantages to nutrient transfer in equids and may have evolved in response to reduced nutrient availability in less invasive equid placentas (figure 2*d–f*). If so, our results may challenge the assumption that epitheliochorial placentas are intrinsically ‘less efficient’ at nutrient transfer than more invasive haemochorial placentas and stress the need for resolving placental structures quantitatively across scales. Our study also suggests that qualitative categories for convergently evolved structures in placental biology, such as ‘villi’, may mask hidden biological variation and adaptation. Although in this study numbers of individual equid species were too low to identify intrageneric differences, our methods could also be used in the future to identify differences in the villi between equid species to interrogate these patterns further.

Villous surface area is important in placental nutrient transfer, with decreases in surface area linked to human placental dysfunctions such as pre-eclampsia [29] and anaemia [30]. As a transporting epithelium, the apical and basal membranes of placental villous trophoblasts contain high densities of transporter proteins and are involved in active transport [31]. Therefore, our finding that equid villi have a higher surface area than human villi for a given volume may confer advantages to nutrient transfer. Likewise, the basal invagination and higher blood vessel indentation of the trophoblast seen in equids could provide benefits to basal transfer through moving nutrients into fetal circulation. Indeed, the high blood vessel indentation of epitheliochorial trophoblasts has before been qualitatively noted [32–35]. Modelling of oxygen versus amino acid (simple versus transporter-mediated) diffusion through these structures highlights the potential benefit that basal trophoblast invaginations could confer by ensuring transported solutes are delivered in close proximity to fetal capillaries, reducing diffusion distances and providing additional surface area for membrane transport. While the basal protrusions may facilitate transfer of substrates requiring membrane transport, it is not clear that they will benefit solutes transferred by simple diffusion. That said, the higher SA : V seen in equid villi compared to humans from our microCT data could confer benefits to oxygen transfer through reduced relative diffusion distances to capillaries and the villus centre from the villus exterior.

If these structural adaptations in equid villi are a response to reduced nutrient availability through decreased invasiveness into maternal tissue as we propose, our data could provide evidence supporting the ‘maternal–fetal conflict hypothesis' in placental evolution. Experimental evidence suggests that placental invasiveness is regulated by the maternal endometrium, such as through the activity of stromal cells, conferring uterine permissiveness [16,36–39]. According to the maternal–fetal conflict hypothesis, epitheliochorial placentation evolved as a way for the mother to constrain nutrients from the fetus in species with precocial energy-intensive young, whereas the genes controlling placental development may be selected for optimal nutrient extraction, potentially at the expense of the mother's long-term fitness interests. We propose, in accordance with the same hypothesis, that as a result fetal placental villi are pressured to compensate for reduced invasiveness through the evolution of structural adaptations to boost nutrient transfer, demonstrated by our data.

For our hypothesis to be fully supported, further work is needed. Our data highlight the limitations of grouping mammalian placentas into qualitative types and stress the need for multiscale quantitative structural characterization. Structures involved in nutrient transfer in mammalian placentas must also be quantified at other scales. An example of why multiscale analysis is necessary is that while this study finds increased surface area of the basal membrane in horses, use of high-resolution volume EM has identified a different adaptation, basal membrane folds, which increase basal membrane surface area in humans at a scale that is not apparent in the current study [40]. The extent to which similar nanoscale infolding boosts exchange area in equids is unknown, however future investigations should include this metric in a holistic understanding of surface area.

Quantifying exchange areas at the scale of gross morphology would also be particularly informative, as it has been qualitatively observed that there seems to be an inverse correlation between gross morphology and invasiveness, with less invasive placentas occupying a greater relative area of the uterine wall [7,8]. This observation is also true in this study, with haemochorial human placentas being discoid (low relative uterine coverage) and epitheliochorial equid placentas being diffuse (high relative uterine coverage) [41]. Is this inverse correlation also a response to reduced placental invasiveness? While future studies are necessary, we propose that our data provide evidence that epitheliochorial placental villi do exhibit structural adaptations to boost nutrient transfer in response to reduced invasiveness.

## 5. Conclusion

The mammalian placenta exhibits massive structural diversity, despite having a single evolutionary origin and conserved functions. Mammalian placentas are currently grouped into qualitative ‘types’, which may hide structural variation important for understanding function, evolution and life-history strategy. Here, we used microCT and EM to quantify structures in placental villi from humans and equids, which have evolved independently and show different levels of invasiveness into maternal tissue. Our study reveals that equid placental villi have a higher SA : V and closer association between the trophoblast basal membrane and the capillary than human villi. We propose that these are structural adaptations to boost nutrient transfer and compensate for reduced invasiveness into maternal tissue in equid placentas. Although further work is needed to fully resolve this hypothesis, these data suggest that mammalian placentas can structurally adapt to boost nutrient transfer efficiency in response to the evolution of reduced maternal nutrient availability and show that not all placental villi are necessarily the same.

## Data Availability

All data (microCT and EM image datasets with segmented labels (.tiff), modelling datasets (.mph), morphometric data (.csv), and metadata (.csv)) associated with this study are freely available to download at Bioimage Archive accession S-BIAD999 [42] under license CC BY 4.0: https://www.ebi.ac.uk/biostudies/bioimages/studies/S-BIAD999. Details of image processing and statistical analysis can be found in the Methods section of this paper.
